# Visualisation of *Kiss1* Neurone Distribution Using a Kiss1‐CRE Transgenic Mouse

**DOI:** 10.1111/jne.12435

**Published:** 2016-10-28

**Authors:** S.‐H. Yeo, V. Kyle, P. G. Morris, S. Jackman, L. C. Sinnett‐Smith, M. Schacker, C. Chen, W. H. Colledge

**Affiliations:** ^1^ Reproductive Physiology GroupDepartment of Physiology, Development and NeuroscienceUniversity of CambridgeCambridgeUK; ^2^ School of Biomedical SciencesUniversity of QueenslandSt LuciaAustralia

**Keywords:** Kiss‐CRE, transgenic, mouse, tdTomato, CLARITY, neuronal distribution

## Abstract

Kisspeptin neuropeptides are encoded by the *Kiss1* gene and play a critical role in the regulation of the mammalian reproductive axis. *Kiss1* neurones are found in two locations in the rodent hypothalamus: one in the arcuate nucleus (ARC) and another in the RP3V region, which includes the anteroventral periventricular nucleus (AVPV). Detailed mapping of the fibre distribution of *Kiss1* neurones will help with our understanding of the action of these neurones in other regions of the brain. We have generated a transgenic mouse in which the *Kiss1* coding region is disrupted by a CRE‐GFP transgene so that expression of the CRE recombinase protein is driven from the *Kiss1* promoter. As expected, mutant mice of both sexes are sterile with hypogonadotrophic hypogonadism and do not show the normal rise in luteinising hormone after gonadectomy. Mutant female mice do not develop mature Graafian follicles or form corpora lutea consistent with ovulatory failure. Mutant male mice have low blood testosterone levels and impaired spermatogenesis beyond the meiosis stage. Breeding Kiss‐CRE heterozygous mice with CRE‐activated tdTomato reporter mice allows fluorescence visualisation of *Kiss1* neurones in brain slices. Approximately 80‐90% of tdTomato positive neurones in the ARC were co‐labelled with kisspeptin and expression of tdTomato in the AVPV region was sexually dimorphic, with higher expression in females than males. A small number of tdTomato‐labelled neurones was also found in other locations, including the lateral septum, the anterodorsal preoptic nucleus, the amygdala, the dorsomedial and ventromedial hypothalamic nuclei, the periaquaductal grey, and the mammillary nucleus. Three dimensional visualisation of *Kiss1* neurones and fibres by CLARITY processing of whole brains showed an increase in ARC expression during puberty and higher numbers of *Kiss1* neurones in the caudal region of the ARC compared to the rostral region. ARC *Kiss1* neurones sent fibre projections to several hypothalamic regions, including rostrally to the periventricular and pre‐optic areas and to the lateral hypothalamus.

Kisspeptins are a set of overlapping neuropeptides that are required for activation of the mammalian reproductive axis at puberty and maintenance of fertility in adults 1, 2. In mice, kisspeptins are derived from a 126 amino acid precursor protein to give shorter peptides of 52, 14, 13 and 10 amino acids that all contain the same carboxy‐terminal sequence. They have a potent action on gonadotrophin‐releasing hormone (GnRH) neurones to stimulate GnRH release and thereby the secretion of follicle‐stimulating hormone and luteinising hormone (LH) from the anterior pituitary. Kisspeptins not only mediate the basal release of GnRH, but also are required for increasing GnRH output to stimulate the LH surge required for ovulation 3, 4, 5. Kisspeptins also regulate seasonal breeding 6 and may act as transducers of peripheral signals to co‐ordinate fertility with metabolic status 7.

Kisspeptins are produced by *Kiss1* neurones, which are found in two distinct regions of the rodent hypothalamus: the arcuate nucleus (ARC) and the RP3V region containing the anteroventral periventricular nucleus (AVPV) and the periventricular preoptic nucleus (PVpo). Kisspeptin expression in the rostral periventricular area of the third ventricle (RP3V) is sexually dimorphic with higher numbers of *Kiss1* neurones in females and it is assumed that these are required for the pre‐ovulatory LH surge 3, 4, 5.

The ability of *Kiss1* neurones to monitor a variety of environmental, metabolic and physiological cues, as well as integrate this information to modulate GnRH secretion, indicates that a complex neural circuitry must exist in the hypothalamus. *Kiss1* neurones in the RP3V region project to GnRH neurone cell bodies, whereas ARC *Kiss1* neurones project to GnRH nerve terminals in the median eminence 8, 9. GnRH neurones express the kisspeptin receptor and respond to kisspeptins with GnRH release. To allow us to begin to map these neural connections, it is necessary to be able to label *Kiss1* neurones in such a way that enables easy visualisation of cell bodies and fibres, ideally in whole tissues. One genetic approach is to express a CRE recombinase specifically in *Kiss1* neurones and then use this to activate a fluorescent reporter protein after a CRE/LoxP‐mediated recombination event.

We have generated a Kiss‐CRE transgenic mouse line in which CRE expression is driven from the *Kiss1* promoter. Homozygous mutant mice lack *Kiss1* expression and are sterile, whereas heterozygous mice are fertile and have been used to activate a tdTomato reporter gene specifically in *Kiss1* neurones for neuronal mapping.

## Materials and methods

### Generation of *Kiss1* ^*tm2(Cre‐GFP)Coll*^ mice

Kiss‐Cre:GFP mice were generated by gene targeting using 129S6Sv/Ev CCB mouse embryonic stem (ES) cells. The targeting vector (pKiss1Cre:GFP) was made by a three‐way ligation using a *Not*I*/Asc*I fragment with 5.7 kb of homology to the *Kiss1* gene from the pKiss1KO plasmid 10, a *Pac*I*/Asc*I *Neo* gene fragment from pTK5IBLMNL (Paradigm Therapeutic, Cambridge, Ltd, UK) and a *Not*I/*Pac*I fragment containing the Cre:GFP coding sequence amplified by a polymerase chain reaction (PCR) from the pCAG‐Cre:GFP plasmid 11. The primers used to amplify the Cre:GFP coding sequence introduced the *Not*I (ACGT*GCGGCCGC*T**ATG**GCCAATTTACTGACCGTACAC) and *Pac*I (ACGT*TTAATTAA*GAGAAGAGGGACAGCTATGAC) restriction sites into the fragment. The *Not*I primer was designed to ensure that the ATG of the *Cre* gene was kept in frame with the coding sequence of the *Kiss1* sequence. After ligation, the ATG of the CRE coding sequence was located 11 codons downstream of the *Kiss1* ATG (**ATG**ATCTCAATGGCTGCGGCCGCT**ATG**GCCAAT—). The translated protein contains the N‐terminal five amino acids of kisspeptin (Met‐Ile‐Ser‐Met‐Ala) and a spacer (Ala‐Ala‐Ala) from the *Not*I site before the Cre‐GFP protein sequence. The gene targeting vector was sequenced to confirm the correct structure.

ES cells containing the targeted *Kiss1* allele were injected into C57Bl/6 host blastocysts to generate male chimeras, which were mated with 129S6Sv/Ev female mice to transmit the targeted alleles to offspring. Mice were genotyped using a multiplex PCR designed to amplify a 320‐bp product specific to the wild‐type allele and a 450‐bp region specific to the *Kiss1* KO allele. All genotypes were observed at the expected Mendelian ratios.

Primers for the wild‐type allele were: mKiss hetF3: CCG TCA TCC AGC CTA AGT TTC TCA C and mKiss hetR3: ATA GGT GGC GAC ACA GAG GAG AAG C.

Primers for the mutant allele were: mKiss a526: GCT TTT ATT GCA CAA GTC TAG AAG CTC and Asc403: CAG CCG AAC TGT TCG CCA GGC TCA AGG.

The line was maintained as heterozygous breeding pairs on a 129S6Sv/Ev inbred genetic background and all animal experiments were approved by a Local Ethics Committee at the University of Cambridge and performed under authority of a Home Office Licence (UK). The official nomenclature for the mice is 129S6‐*Kiss1* ^*tm2(Cre‐GFP)Coll*^. To visualise *Kiss1* neurones, the *Kiss1* ^*tm2(Cre‐GFP)Coll*^ mice were bred with *B6;129S6‐Gt(ROSA)26Sor* ^*tm9(CAG‐tdTomato)Hze*^ reporter mice (Strain no. 007905; Jackson Laboratories, Bar Harbor, Maine, USA, which have a *loxP*‐flanked STOP cassette preventing transcription of a CAG promoter‐driven red fluorescent protein variant (tdTomato). TdTomato is expressed following Cre‐mediated recombination.

### Hormone assays

Plasma hormone levels were measured by an enzyme‐linked immunosorbent assay (ELISA). Blood was collected in a syringe containing 2 μl of 0.5 M ethylenediaminetetraacetic acid from the inferior vena cava and centrifuged at 10 000 ***g*** for 15 min at 4 °C. The plasma was collected and stored at −80 °C until assayed. LH was measured using an in‐house ELISA as described by Steyn *et al*. 12. In our hands, the sensitivity of this ELISA is 10 pg/ml with an intra‐assay variation of 4.29% and an inter‐assay variation of 6.05%. Testosterone was measured by using an ELISA kit (EIA 1559; DRG Diagnostics, Marburg, Germany) with a sensitivity of 0.16 ng/ml (intra‐assay variation, 4.16%; inter‐assay variation, 9.94%). Follicle‐stimulating hormone was measured using a colourimetric ELISA kit (ERK7007; Endocrine Technologies, Newark, NJ, USA) with a sensitivity of 0.5 ng/ml and intra‐assay variation of 6.45% and an inter‐assay variation of 7.11%.

### Tissue preparation

Unless stated otherwise, all chemicals and reagents were purchased form Sigma‐Aldrich (Poole, UK). For histology, tissues were fixed in 4% paraformaldehyde/phosphate‐buffered saline (PBS) overnight at 4 °C, dehydrated through graded alcohols, wax embedded and 7‐mm sections were stained with haematoxylin and eosin. To examine co‐labelling of kisspeptin and tdTomato, mice were gonadectomised to facilitate visualisation of kisspeptin‐immunoreactive cell bodies in the ARC 13. The animals were allowed to recover for a period of 12 days before perfusion. Mice were anaesthetised with an overdose of pentobarbital (3 mg per 100 μl) and perfused transcardially with 15 ml of 4% paraformaldehyde (PFA) in 0.1 m PBS at pH 7.6. The brains were removed, post‐fixed in the same fixative at room temperature (RT) for 1 h and then transferred to 30% sucrose/Tris‐buffered saline (TBS) (50 mm Tris, pH 7.6, 0.8% NaCl) for cryoprotection. Three sets of 40‐μm coronal brain sections were cut from the level of the medial septum through to the hindbrain for free‐floating immunohistochemistry (IHC). For mapping of tdTomato expression, 100‐μm slices were prepared using a vibratome (VT1000; Leica Microsystems, Wetzlar, Germany) and mounted on slides.

### Fluorescence IHC

Immunofluorescence labelling was performed on free‐floating coronal brain sections. For kisspeptin and tdTomato immunostaining, sections were incubated overnight with a sheep anti‐kisspeptin‐10 antiserum (AC024, dilution 1 : 2000; gift from Alain Caraty) combined with a rabbit anti‐RFP antibody (dilution 1 : 2000; Rockland Immunochemicals, Pottstown, PA, USA) in TBS containing 2% normal donkey serum, 0.3% Triton‐X‐100 and 0.25% bovine serum albumin. After several washes with TBS, the sections were placed in biotinylated donkey anti‐sheep secondary immunoglobulins (dilution 1 : 200; Jackson ImmunoResearch, West Grove, PA, USA) and then incubated with a combination of Alexa Fluor 488‐conjugated strepavidin and Alexa Fluor 568‐conjugated goat anti‐rabbit immunoglobulins, each for 90 min at RT (dilution 1 : 200; Molecular Probes, Carlsbad, CA, USA). All sections were then washed, mounted on slides, air dried, and cover slipped with Vectashield Fluorescence Mounting Medium (Vector Laboratories, Inc., Burlingame, CA, USA). Controls consisted of the omission of primary and/or secondary antibodies for the different combinations, and these sections consistently failed to exhibit the appropriate immunofluorescence.

### Image analysis

All images were generated using a TCS SP2 Laser Scanning Confocal Microscope (Leica Microsystems) at the Cambridge Advanced Imaging Centre (Cambridge, UK). Images were captured using a × 63 oil/glycerine/water‐immersion objective (numerical aperture of 1.20: working distance 300 μm). Alexa Fluor 488 and tdTomato (568) were excited with 488 and 561 laser lines and emission collected with 500–550‐nm and 580–620‐nm bandpass emission filters, respectively. All images were captured using sequential scanning mode and image stacks were collected at focus intervals of 1.0 μm. Eight‐bit confocal images were acquired with a 512 × 512 pixels format and a scan speed of 400 Hz. All images were digitally processed in Photoshop (Adobe Systems Inc., San Jose, CA, USA), where the levels of brightness and contrast were adjusted to enhance the quality of images. The number of tdTomato and kisspeptin‐stained neurones was counted in each region of two brain sections for each animal (mean ± SE).

### CLARITY method

The CLARITY method was based on protocols reported by Chung and Deisseroth 14. Briefly, mice were killed by injection of pentobarbital, and immediately perfused transcardially with 20 ml of ice‐cold PBS followed by 20 ml of ice‐cold hydrogel solution. The brain was removed and placed into 20 ml of ice‐cold hydrogel solution for incubation in the dark at 4 °C for 4 days. Polymerisation was initiated by incubation at 37 °C for 3.5 h. Samples were initially cleared by passive clearing followed by electrophoresis. For passive clearing, the samples were placed in clearing solution for 72 h at 37 °C, with the solution being changed twice every 24 h. For clearing, the samples were subjected to electrophoresis at 25 V for 3 days with circulating clearing buffer at 30 °C. After clearing, the samples were placed in 1 × PBS containing 1% Triton X‐100 and washed for 48 h on a slowly rotating shaker with the solution being changed every 12 h. The sample was stored in PBST until 24 h before imaging, and then transferred into FocusClear medium (CelExplorer Labs, Hsinchu City, Taiwan). After 24 h, the sample and surrounding medium should have the same refractive index. Imaging was performed using an Axio Zoom V16 microscope combined with Apotome.2 (Carl Zeiss Ltd, Cambridge, UK) for wide field imaging and optical sectioning of the cleared brain.

The hydrogel solution was prepared as a 1 × PBS solution containing: 4% acrylamide (Bio‐Rad, Hercules, CA, USA), 0.05% bis‐acrylamide (Bio‐Rad), 4% PFA (Electron Microscopy Sciences, Hatfield, PA, USA) and 2.5 mg/ml of the VA‐044 initiator (2,2′‐azobis[2‐(2‐imidazolin‐2‐yl)propane]dihydrochloride) (Wako Pure Chemical Industries, Ltd, Osaka, Japan). Clearing solution was prepared at 50 °C with 200 mm boric acid and 4% sodium dodecyl sulphate (Fisher Scientific Co., Pittsburgh, PA, USA) with the pH adjusted to 8.5 using 1 m NaOH.

### Quantification of tdTomato‐expressing cells in CLARITY brains

All images were captured using the × 2.3 Plan‐NEOFLUAR Z lens with a numerical aperture of 0.57 in an object field of 1.5 mm. Optical sections of 1.5 μm for each stack were corrected with Apotome.2 for high contrast and best resolution. Three‐dimensional images were constructed using the 3D reconstruction feature of zen software (Carl Zeiss Ltd). Quantification was performed using fiji/imagej (NIH, Bethesda, MD, USA) using 2D stacked images. All images were converted into eight‐bit binary mode, subjected to background reduction and outlier removal, and then watershed segmentation prior to particle analysis.

### Immunostaining for tdTomato‐expressing cells and fibres and optical clearing using 2,2′‐thiodiethanol (TDE) reagent

The TDE clearing protocol was modified from a published method by Gonzalez‐Bellido and Wardill 15. Female mice at postnatal day 33 (P33) and day 56 (P56) were perfused with 4% PFA and the brains extracted. The brains were sliced using a vibratome into 1‐mm thick horizontal slices. The slices were washed in PBS and treated with 10 mm copper sulphate in 50 mm ammonium acetate (pH 5.0) for 12 h at 4 °C for autofluorescence quenching. After rinsing in PBS, the slices were blocked with 10% goat serum for 45 min at RT. The slices then underwent serial alcohol dehydration and rehydration steps in 50%, 70%, 90% and 100% ethanol. The slices were permeated using a cocktail of collagenase (0.5 mg/ml) and dispase (300 μg/ml) in PBS at 37 °C for 45 min. Slices were washed overnight and incubated with primary antibody rabbit anti‐RFP (dilution 1 : 600, Rockland Immunochemicals) added with 2% goat serum for 72 h at 4 °C. The slices were incubated with secondary immunoglobulin–goat anti‐rabbit conjugated with DyLight 568 (dilution 1 : 200; Thermo Scientific, Waltham, MA, USA) for 72 h at 4 °C. Prior to TDE optical clearing, slices were washed overnight in PBS, and then incubated at RT in an increasing concentration of TDE solutions; 1 h for each concentration, 10%, 20%, 40%, 60%, 80%, 90% and 97% TDE (w/v%), in PBS. Finally, slices were immersed in imaging chambers containing 97% TDE, which matches the refractive index of oil, and were imaged immediately using a confocal microscope (SP2; Leica; Cambridge Advanced Imaging Centre). A series of images was captured with a × 20 oil/glycerin/water‐immersion objective (numerical aperture of 0.75; working distance 680 μm) at optical sections of 0.8 μm across the hypothalamus of the horizontal slices. For 2D reconstruction of all the hypothalamic image stacks, the images were stitched using fiji/imagej.

### Statistical analysis

The statistical tests used are indicated as appropriate. For data sets that did not pass a normality test, a nonparametric test was used. If an anova test gave P < 0.05, a multiple comparison post‐test was performed to determine which pairs were responsible for the significant deviation. P < 0.05 was considered statistically significant in post‐test comparisons.

## Results

**Figure 1:**
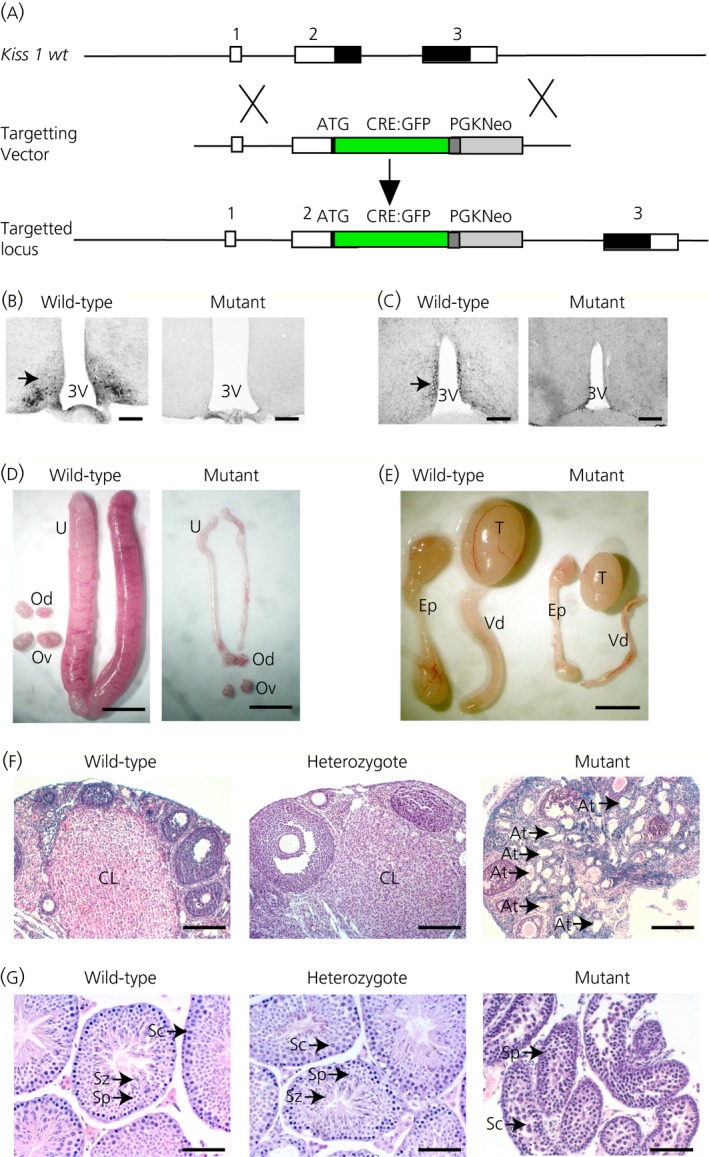
Generation of *Kiss1* ^*tm1(Cre‐*^ ^*GFP*^ ^*)Coll*^ mutant mice and hypogonadism. (a) Gene targeting strategy. The *Kiss1* gene consists of three exons with the coding regions shown in black. The targeting vector contains a CRE‐GFP transgene immediately downstream of the *Kiss1* initiation codon so that CRE expression is regulated by the *Kiss1* promoter. (b, c) Confirmation of a null mutation by immunocytochemistry for kisspeptin expression (arrowed) in the arcuate nucleus (b) and the anteroventral periventricular nucleus (AVPV) (c) regions of the hypothalamus. Scale bar = 150 μm. (d) Female reproductive organs showing thread‐like uteri in mutants. U, uterus; Od, oviduct; Ov, ovary. Scale bar = 0.5 cm. (e) Male reproductive organs showing reduced growth. T, testes; Ep, epididymis; Vd, vas deferens. Scale bar = 1 cm. (f) Histology of ovaries showing corpora lutea (CL) in wild‐type and heterozygous mice but no CL in the mutant mice. Large numbers of atretic follicles (At, arrows) were found in the mutant mice. Scale bar = 200 μm. (g) Histology of testes showing spermatozoa (Sz) in the seminiferous tubules of wild‐type and heterozygous mice but none in the mutant testes. Sc, spermatocytes; Sp, spermatids. Scale bar = 100 μm.

Gene targeting in mouse ES cells was used to insert a CRE‐GFP transgene immediately downstream of the ATG initiation codon in exon 2 of the *Kiss1* gene in frame with the *Kiss1* coding sequence so that expression is driven from the endogenous *Kiss1* promoter (Fig. 1 a). The insertion disrupts the *Kiss1* coding sequence so that mutant mice do not produce the kisspeptin neuropeptide. Immunohistochemisty did not detect any kisspeptin protein in the ARC or the RP3V hypothalamic regions of mutant mice compared to strong staining in wild‐type mice (Fig. 1 b). These data confirm that the *Kiss1* ^*CRE‐GFP*^ allele is a null mutation.

Mutant male mice had a lower body weight than age‐matched control mice, whereas females did not demonstrate any difference between the two genotypes (Table 1). Heterozygous mice were fertile and showed normal fecundity compared to wild‐type mice with similar reproductive tissue weights (Table 1) and histology (Fig. 1), as well as time to the birth of the first litter (see Supporting information, Fig. S1a) and litter sizes (see Supporting information, Fig. S1b). By contrast, male and female homozygous mutant mice were sterile. Mutant female mice showed lower weights of their ovaries and uteri (Fig. 1 d and Table 1). Mutant male mice had significantly lower weights for their testes, epididymis, vas deferens and kidneys (Fig. 1 e and Table 1). Histological analysis of the ovaries of adult mutant mice did not show any Graaffian follicles or corpora lutea indicating failure of ovulation and the mutant ovaries had large numbers of atretic follicles (Fig. 1 f, arrowed). *Kiss1* ^*CRE‐GFP*^ null male mice around 2 months of age failed to complete spermatogenesis, with arrest just after meiosis at the round spermatid stage and with very few sperm in the seminiferous tubules (Fig. 1 g). Around 4–5 months of age, however, condensed sperm heads were found in mutant mice, indicating an increased level of spermiogenesis in older males. We have observed a similar increase previously in *Kiss1* KO mice, which we have shown to be a result of exposure to phyto‐oestrogens in the mouse chow 16.

**Table 1:** Organ Weights (g) in Mutant Mice

Males	Body	Testes*	Epididymis*	Vas deferens*	Kidney*
Wild‐type (n = 10)	30.8 ± 0.37	0.090 ± 0.008	0.034 ± 0.003	0.0093 ± 0.003	0.24 ± 0.006
Heterozygote (n = 5)	31.9 ± 0.90	0.107 ± 0.013	0.038 ± 0.004	0.0088 ± 0.0010	0.25 ± 0.035
Mutant (n = 10)	**26.0 ± 0.76** **a**	**0.037 ± 0.004** **c**	**0.014 ± 0.005** **d**	**0.0031 ± 0.0005** **e**	**0.14 ± 0.006** **b**

Females	Body	Ovary	Oviduct	Uterus
Wild‐type (n = 10)	24.4 ± 0.52	0.0060 ± 0.0004	0.0020 ± 0.0004	0.099 ± 0.016
Heterozygote (n = 4)	25.1 ± 0.48	0.0075 ± 0.0003	0.0025 ± 0.0003	0.062 ± 0.005
Mutant (n = 12)	25.4 ± 0.52	**0.0015 ± 0.0004** **f**	0.0015 ± 0.0004	**0.007 ± 0.004** **g**

Mice were killed at 5 months of age and the organs weighed. Significant differences between wild‐type and mutant mice (shown in bold) were found in tissues of the reproductive system. No significant differences were found between wild‐type and heterozygous mice. Values are given as the the mean ± SE. *Values corrected for weight difference between wild‐type and mutant mice (/weight × 25 g). ^a^P < 0.001 versus wild‐type (two‐tailed, t‐test). ^b^P < 0.0001 versus wild‐type (two‐tailed, t‐test). ^c^P < 0.001 versus wild‐type (two‐tailed, t‐test). ^d^P < 0.001 versus wild‐type (two‐tailed, Mann–Whitney test). ^e^P < 0.0001 versus wild‐type (two‐tailed, Mann–Whitney test). ^f^P < 0.001 versus wild‐type (two‐tailed, Mann–Whitney test). ^g^P < 0.0001 versus wild‐type (two‐tailed, Mann–Whitney test).

**Figure 2:**
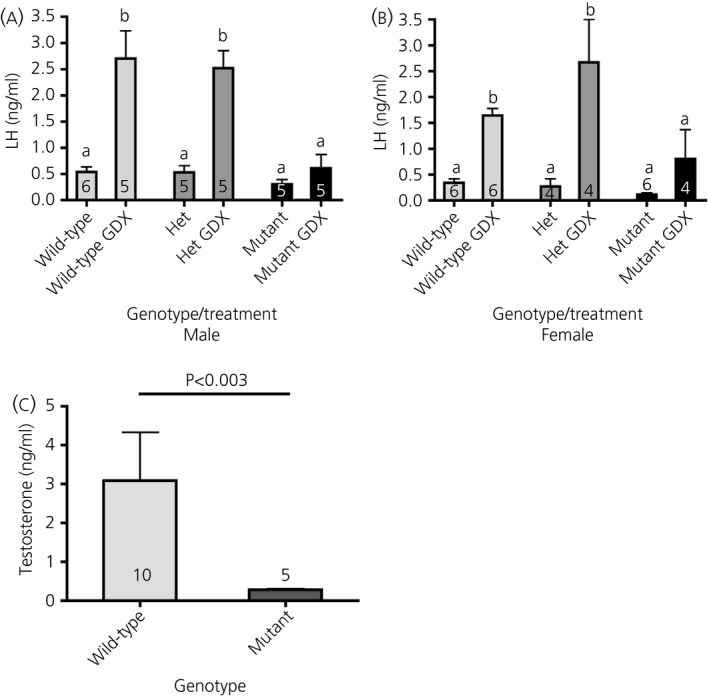
Hormone profiles of *Kiss1* ^*tm1(Cre‐*^ ^*GFP*^ ^*)Coll*^ mutant mice. Blood plasma luteinsing hormone (LH) levels in males (a) and females (b). No difference in basal LH was found between wild‐type and mutant mice, although mutant mice failed to show the post‐gonadectomy (GDX) rise in LH. Data were analysed by a nonparametric anova (Kruskal–Wallis test, P < 0.005) with a Dunn's multiple comparison post‐test. (c) Blood plasma testosterone levels were significantly reduced in mutant mice. The data were analysed by a nonparametric Mann–Whitney test. The number of mice in each group is indicated on the histogram.

There was no significant difference in basal LH between the genotypes. After gonadectomy, however, the wild‐type mice showed the expected rise in LH, whereas the mutant mice did not (Fig. 2 a,b). Heterozygous mice showed a magnitude in rise in LH similar to that of wild‐type mice (Fig. 2 a,b). Mutant male mice had significantly lower blood testosterone levels compared to wild‐type mice (Fig. 2 c).

**Figure 3:**
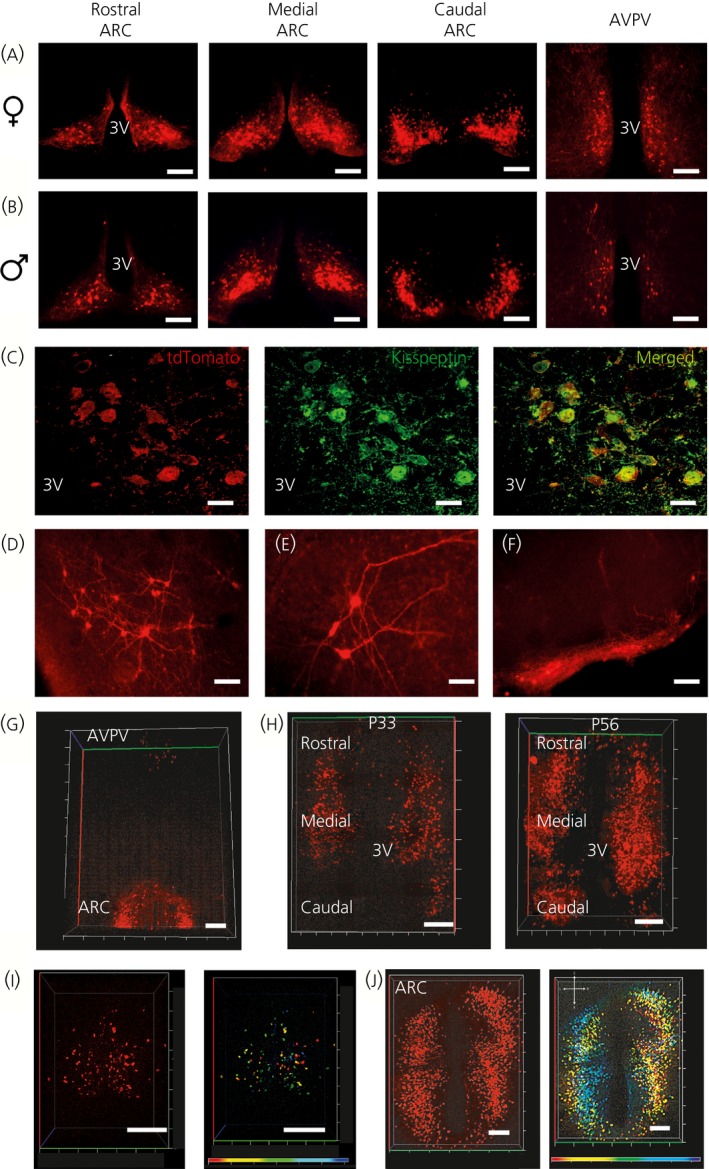
Fluorescent visualisation of *Kiss1* neurones. *Kiss1* neurones were visualised by breeding Kiss‐CRE mice with floxed tdTomato mice *Gt(ROSA)26Sor* ^*tm9(*^ ^*CAG*^ ^*‐tdTomato)Hze)*^. (a) Fluorescent *Kiss1* neurones in the arcuate nucleus (ARC) and the anteroventral periventricular nucleus (AVPV) regions of females. (b) Fluorescent *Kiss1* neurones in ARC and AVPV regions of males. (c) Co‐localisation of tdTomato neurones with kisspeptin protein expression in the ARC (gonadectomised mouse). Scale bar = 10 μm. (d) Lateral septum. Scale bar = 50 μm. (e) Amygdala. Scale bar = 25 μm (f) Mammillary nucleus. Scale bar = 50 μm. (g) CLARITY image showing *Kiss1* neurone distribution in rostral ARC and AVPV regions of female mice. (h) CLARITY images showing the increase in the number of fluorescent *Kiss1* neurones in peri‐pubertal (post‐natal day 33; P33) and post‐pubertal (post‐natal day 56; P56) mice. (i). CLARITY image of the AVPV region in a female mouse and a false colour image to show depth of *Kiss1* cells. (j) CLARITY image of the ARC region in a female mouse and false colour image to show depth of *Kiss1* cells. 3V, third ventricle; ARC, arcuate; AVPV, anteroventral periventricular nucleus. All scale bars in CLARITY images = 200 μm.

Although the GFP gene has been inserted into the *Kiss1* gene, we did not detect any green fluorescent neurones in heterozygous or homozygous transgenic mice. To visualise expression of the CRE recombinase protein, the *Kiss1* ^*CRE‐GFP*^ mice were bred with a reporter line (*Gt(ROSA)26Sor* ^*tm9(CAG‐tdTomato)Hze*^) in which a CAG‐tdTomato transgene is activated by CRE. Strong tdTomato expression was found extending from the rostral to the caudal parts of the ARC region of the hypothalamus and in both the AVPV and the PVpo of the RP3V region (Fig. 3 a,b and Table 2). The number of tdTomato‐labelled neurones in the RP3V region showed a clear sexual dimorphism, with approximately twice as many in females compared to males (Fig. 3 a,b and Table 2). The number of tdTomato‐labelled neurones in the ARC was similar between the sexes and both sexes showed increase in the number between the rostral and caudal regions (Table 2). These data are consistent with tdTomato expression being restricted to *Kiss1* neurones. To determine whether tdTomato expression specifically labelled *Kiss1* neurones, we performed IHC with an anti‐kisspeptin antibody for co‐localisation between the tdTomato protein and kisspeptin (Fig. 3 c). Visualisation of *Kiss1* neurone cell bodies is difficult in the ARC region because of the high density of kisspeptin fibres in this region. To allow better visualisation of kisspeptin‐expressing cell bodies in the ARC, we gonadectomised mice before IHC. The percentage of tdTomato neurones in the ARC co‐expressing kisspeptin was between 81% and 90% in males and between 85% and 91% in females (Table 2). In the RP3V region, the percentage of tdTomato neurones co‐expressing kisspeptin was 31–45% in males and 76–83% in females (Table 2). Conversely, approximately 70–80% of Kiss1 neurones in the RP3V region expressed tdTomato and 95–100% of Kiss1 neurones in the ARC expressed tdTomato.

**Table 2:** Quantitation of tdTomato‐Expressing Neurones Co‐labelled with kisspeptin

Hypothalamic areas	Male GDX (n = 4)				Female GDX (n = 3)			
	Number of tdTomato neurones	Number of kisspeptin neurones	% tdTomato neurones expressing kisspeptin	% kisspeptin neurones expressing tdTomato	Number of tdTomato neurones	Number of kisspeptin neurones	% tdTomato neurones expressing kisspeptin	% kisspeptin neurones expressing tdTomato
AVPV	15 ± 2	7 ± 1	31 ± 2	67 ± 4	24 ± 3	23 ± 3	76 ± 5	79 ± 6
PVpo	18 ± 1	11 ± 1	45 ± 3	73 ± 3	44 ± 8	43 ± 10	83 ± 5	84 ± 3
Rostral ARC	62 ± 8	57 ± 9	90 ± 3	99 ± 1	41 ± 6	37 ± 7	90 ± 4	99 ± 1
Middle ARC	105 ± 5	99 ± 7	90 ± 4	96 ± 1	116 ± 13	106 ± 11	91 ± 1	99 ± 1
Caudal ARC	174 ± 19	152 ± 18	81 ± 5	92 ± 3	149 ± 18	135 ± 17	85 ± 2	95 ± 3

Mice were gonadectomised and killed after 14 days for fluorescent visualisation of tdTomato‐expressing neurones and immunohistochemical detection of *Kiss1* neurones. AVPV, anteroventral periventricular nucleus; ARC, arcuate nucleus; PVpo, periventricular preoptic nucleus.

**Figure 4:**
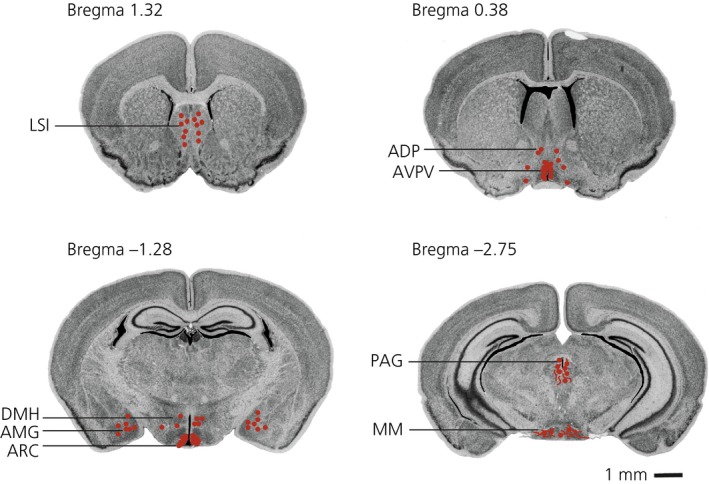
Distribution of Kiss‐CRE/tdTomato labelled neurones in the female mouse brain. Coronal brain images were taken from the online Mouse Brain Library resource 30. TdTomato positive cell bodies are represented by red circles and fibres represented by red lines. LSI, lateral septum, intermediate; ADP, anterodorsal preoptic nucleus; AVPV, anteroventral periventricular nucleus; AMG, amygdala; DMH, dorsomedial hypothalamus; ARC, arcuate nucleus; PAG, periaquaductal grey; MM, medial mammillary nucleus.

A detailed analysis of tdTomato expression in thick serial sections throughout the brain identified several regions outside the RP3V and the ARC where tdTomato activation was found in neuronal cell bodies (Figs 3 d–f and 4). These regions included the lateral septum (Fig. 3 d), the anterodorsal preoptic nucleus, the amygdala (Fig. 3 e), the medial preoptic nucleus, the anterior hypothalamic area, the dosomedial and ventromedial hypothalamic nuclei, the periaquaductal grey, and the mammillary nucleus (Fig. 3 f). Quantitation of the number of tdTomato positive neurones in these regions indicated similar numbers in both heterozygous and mutant females (Table 3), apart from the amygdala, where mutant mice had almost twice the number (Table 3). The number of cell bodies in the mammillary nucleus was low, although there was a large number of fibres in this region (Fig. 3 f).

**Table 3:** Quantitation of tdTomato‐Expressing Neurones in Other Brain Regions

Genotype	Number of tdTomato‐expressing neurones (two sections per region per animal)				
	Lateral septum	Amygdala	Dorsal medial nucleus	Periaquaductal grey	Mammillary nucleus
Heterozygous females (n = 5)	11 ± 4	13 ± 3	7 ± 2	3 ± 1 (n = 3)	7 ± 8 (n = 3)
Homozygous females (n = 4)	10 ± 5	20 ± 3*	13 ± 2	5 ± 2 (n = 3)	4 ± 3 (n = 3)

TdTomato‐expressing neurones were mapped in other brain regions after vibratome sectioning. The number of mice in each group is indicated in brackets. Two sections per mouse were examined for each region. No difference in the number of tdTomato neurones was found, except in the amygdala where homozygous mutant mice had a significantly greater number compared to wild‐type mice (*P < 0.05, Student's t‐test).

To map the distribution of Kiss1 neurones in three dimensions, we processed brains using the CLARITY method 14, which removes lipids from tissues to render them transparent. Using this technique, we could visualise Kiss1 neurones in the ARC and the AVPV regions simultaneously (Fig. 3 g). We found that there was an increase in the number of tdTomato‐labelled neurones between postnatal day 33 and 56, which is consistent with increasing *Kiss1* promoter activity during puberty (Fig. 3 h). The ARC kisspeptin cell number was significantly higher in P56 brains (1059 ± 37 versus 812 ± 51; P = 0.028; Mann–Whitney test; see Supporting information, Fig. S2). An increase in the number of *Kiss1* tdTomato neurones was also observed in the middle and caudal aspect of the arcuate nucleus after puberty. The CLARITY method also allows processing of images with a false colour overlay to provide a representation of the depth of the neurones in the tissue (Fig. 3 i,j). *Kiss1* neurones in the AVPV region spanned a depth of 300 μM (Fig. 3 i), whereas those in the ARC spanned a narrower depth of 140 μm (Fig. 3 j).

**Figure 5:**
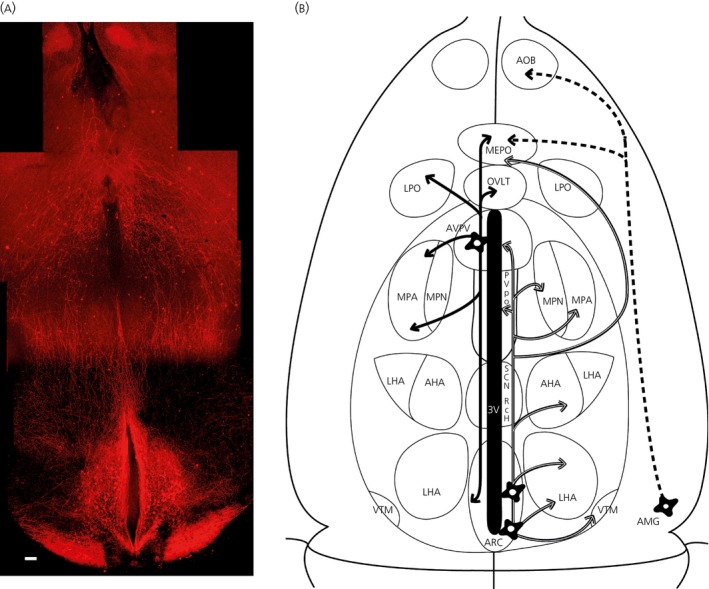
Fibre distribution in the Kiss‐CRE/tdTomato female mouse brain. (a) Montage of Z‐stack series of the ventral hypothalamus of a P56 age mouse brain. Immunocytochemistry of 2,2′‐thiodiethanol (TDE)‐cleared horizontal slices enabled the visualisation of tdTomato fibre projections. Scale bar = 200 μm. (b) Schematic horizontal view of the ventral hypothalamus (approximately the same scale as in a) illustrating the fibre projections from *Kiss1* neurones. Anteroventral periventricular nucleus (AVPV) *Kiss1* neuronal projections are indicated by by solid black arrows. Arcuate nucelus (ARC) *Kiss1* projections are indicated by white arrows. Amygdala *Kiss1* projections are indicated by dotted arrows. 3V, 3rd ventricle; AHA, anterior hypothalamic area; AMG, amygdala; AOB, accessory olfactory bulb; LHA, lateral hypothalamic area; LPO, lateral preoptic area, MEPO, median preoptic nucleus; MPA, medial preoptic area; MPN medial preoptic nucleus; Rch, retrochiasmatic area; SCN, suprachiasmatic area; OVLT, vascular organ of lamina terminalis, PVpo, periventricular preoptic nucleus; VTM, ventral tuberomammillary nucleus.

To allow optimal visualisation of *Kiss1* fibre projections, we performed immunostaining for tdTomato and cleared thin sections of the brains with the TDE reagent. We found fibres extending from the ARC into the lateral hypothalamus; in particular, the caudal ARC *Kiss1* neurones formed a dense fibrous network encompassing the lateral hypothalamic area, the ventral pre‐mammillary nucleus, the ventral tegmental area and the mammillary nucleus (Fig. 5). The medial *Kiss1* neurones also projected into the lateral hypothalamic area, as well as rostrally to the periventricular and preoptic regions, where the fibres segregated into lateral and medial pathways. The regions with very sparse kisspeptin fibres are the retrochiasmatic and the suprachaismatic nuclei. The RP3V *Kiss1* neurones project rostrally to the rostral preoptic area (POA) and laterally to the lateral septum. The RP3V *Kiss1* neurones also send descending fibres through the periventricular region into the ARC. We also found fibre projections from the amygdala into the POA and extending further into the accessory olfactory bulb (AOB) (Fig. 5).

## Discussion

We have generated a transgenic mouse line in which the *Kiss1* gene has been disrupted by the insertion of a CRE:GFP transgene. The phenotype of the mutant mice is as expected from a *Kiss1* null mutation; namely, an absence of sexual maturation and sterility in both sexes. Female mice do not show oestrus cyclicity or ovulate, and have underdeveloped ovaries, oviducts and uteri. Male mice have a microphallus, smaller testes, and underdeveloped epididymides and vas deferens. In young mice, spermatogenesis is arrested at the primary spermatocyte stage prior to meiotic division, whereas older mice show some capacity to complete spermatogenesis probably as a result of exposure to phyto‐oestrogens in their food 16. Neither sex shows the normal rise in LH after gonadectomy indicating a failure to respond to removal of sex steroid negative feedback on the hypothalamus. Male mice also have low testosterone levels in the blood. These data indicate that the mutant mice have hypogonadotrophic hypogonadism similar to other *Kiss1* null mutants 10, 17, 18.

The mutant mice of either sex did not show any difference in basal LH levels compared to wild‐type mice. Although we used a very sensitive assay 12 that can detect LH at concentrations as low as 10 pg/ml, we collected blood at a single time point and our samples did not correspond to any LH peaks, so that the mutant and wild‐type mice showed similar basal levels of LH. Serial blood sampling would be required to detect pulsatile LH in the wild‐type mice, which would be expected to be absent in the Kiss‐CRE mutant mice, similar to lack of LH pulsatility in Gpr54 mutant mice 12. Indeed, serial blood measurements by the group of Prof Allan Herbison indicates no LH pulsatility in the Kiss‐CRE mutant mice (A. Herbison, personal communication). Nevertheless, after gonadectomy and loss of sex steroid negative feedback on the *Kiss1* neurones, wild‐type and heterozygous mice showed a rise in basal LH levels, which did not occur in the Kiss‐CRE mutant mice. This was expected because the mutant mice do not produce any kisspeptin protein with which to stimulate GnRH release.

The lower body weight of the mutant male mice compared to age‐matched wild‐type mice is likely a consequence of the low testosterone levels in the mutants. Testosterone is known to increase muscle mass at puberty and patients with hypogonadotrophic hypogonadism often have lower muscle mass 19. Mice with a disruption of the androgen receptor have a 12–13% reduction in body mass and a decreased muscle mass 20. Androgens are proposed to increase muscle mass by regulating genes involved in controlling the progression of myoblast proliferation to differentiation.

The CRE:GFP gene is in frame with the *Kiss1* coding sequence just downstream of the *Kiss1* initiation codon. Thus, expression of CRE recombinase protein is restricted to neurones in which the *Kiss1* promoter is active. We could not detect GFP fluorescence in *Kiss1* neurones most likely because the *Kiss1* promoter is relatively weak. Thus, to visualise *Kiss1* neurones, we bred the Kiss‐CRE mice with *Rosa26* ^*CAGtdTomato*^ reporter mice in which the tdTomato gene is activated by CRE recombinase. Strong tdTomato expression was found in both the RP3V and ARC regions of the hypothalamus, with the majority (80–90%) of these neurones co‐expressing kisspeptin. An exception to this co‐expression was found in the RP3V region of male mice. This region is sexually dimorphic with fewer *Kiss1* neurones in males compared to females. We found that only 30–45% of tdTomato positive neurones expressed kisspeptin in this region. The reason for this is not known, although it could be *Kiss1* neurones that activated the tdTomato transgene and then turned off *Kiss1* gene expression during the period of masculinisation. Similar co‐expression data in this region in males have been reported for other *Kiss1* reporter mice 21, 22, 23. We have previously determined the distribution of *Kiss1* neurones in the adult female mouse brain by IHC 24. In that study, many kisspeptin cell bodies were found in the RP3V and ARC regions as expected, although smaller numbers of kisspeptin‐expressing neurones were also found in the dorsomedial nucleus (DMN) and posterior hypothalamus. We also found tdTomato‐labelled cells in the DMN and the posterior hypothalamus in the *Kiss1* ^*tm2(Cre‐GFP)Coll*^ mice and so we consider that these are authentic *Kiss1* neurones. Indeed, kisspeptin protein has been detected in soma in the DMN using an antibody (AC566) directed against the Kp10 protein 24, 25.

Other studies have also generated Kiss‐CRE mice 21, 22, 23. Cravo *et al*. 21 generated a Kiss1‐CRE BAC transgenic line (*Tg* ^*(Kiss1‐cre)J2‐4Cfe*^) containing 109 kb of genomic sequence upstream of the *Kiss1* start codon and 69 kb of genomic sequence downstream of the *Kiss1* stop codon. The *Cre* transgene is fused in frame with the *Kiss1* initiation codon, although the rest of the *Kiss1* coding sequence, including the first intron, has been removed. Expression of the Kiss‐CRE BAC transgene was visualised by breeding the mice with GFP or *LacZ* reporter mice. Reporter gene expression was found in approximately 90% of *Kiss1* neurones, identified by *in situ* hybridisation, in the AVPV, PeN and the ARC, although expression was also found in additional brain regions, including the cerebral cortex and the medial nucleus of the amygdala.

A second Kiss‐CRE model (*Kiss1* ^*tm1.1(cre)Uboe*^) generated by Mayer *et al*. 23 via gene targeting in mouse ES cells has an IRES‐CRE transgene downstream of the *Kiss1* stop codon. Kiss‐CRE mediated activation of a fluorescent reporter protein in these mice showed that > 95% of Kiss1 neurones in the ARC or the AVPV were fluorescent 23, whereas 80–90% of GFP positive cells co‐labelled for kisspeptin 26. Again, expression of GFP was found in other brain regions including the cerebral cortex, hippocampus, posterior hypothalamus, periaquaductal gray, premammillary nucleus and the amygdala 26.

A third Kiss‐CRE model (*Kiss1* ^*tm1.1(cre/EGFP)Stei*^) was developed by Gottsch *et al*. 22. This mouse contains a *Cre‐eGfp* transgene targeted immediately upstream of the *Kiss1* coding region. Unlike the other Kiss‐CRE mice, *Kiss1* neurones should be detectable by GFP expression without having to cross these mice with a CRE‐activated reporter mouse. In reality, GFP fluorescence from the targeted transgene was weak and difficult to detect in gonadally intact animals that have sex steroid feedback, possibly reflecting the relative strength of the *Kiss1* promoter. Gottsch *et al*. 22 used GFP IHC to improve visualisation and bred the mice with a *LacZ* ^*LoxP*^ reporter mouse to study co‐localisation of GFP and β‐galactosidase. Approximately 75% of cells were co‐labelled with GFP and β‐galactosidase, with a number of neurones in the ventromedial hypothalamus showing β‐galactosidase expression only.

All published Kiss‐CRE transgenic lines show expression of a CRE‐activated reporter gene (*Gfp* or *LacZ*) at sites where kisspeptin protein is not normally found. Similarly, in our Kiss‐CRE mice, CRE‐activation of a tdTomato reporter gene was found at ectopic sites. Some of these regions are the same as those reported for the other Kiss‐CRE lines including the ventromedial hypothalamic nucleus and the posterior hypothalamus. We also find differences, however, between our Kiss‐CRE mice and these others; in particular, we do not observe reporter gene activation in the cerebral cortex, which has been found in the *Tg* ^*(Kiss1‐cre)J2‐4Cfe*^ and the *Kiss1* ^*tm1.1(cre)Uboe*^ lines. The reason for this difference is not known, although it might be caused by the genomic context of the targeted alleles. Loss or displacement of negative regulatory domains by the CRE transgene could allow the *Kiss1* promoter to be expressed in neuronal populations where this does not normally occur.

There are several possibilities that might explain the ectopic sites of tdTomato expression. First, the tdTomato reporter might reflect *Kiss1* gene expression during development, which results in permanent reporter gene activation in all daughter cells, although *Kiss1* expression is not maintained in these cells in the adult. Another possibility is that some of the ectopic cells with reporter gene expression have *Kiss1* transcription, whereas the mRNA is not translated into protein or it is rapidly degraded. Finally, the level of expression of kisspeptin protein might be below the detection limits of IHC. Although we found tdTomato expression in the amygdala, we could not detect kisspeptin protein (data not shown). This is in contrast to a recent report, where kisspeptin immunoreactivity was found in the amygdala of rats 27. It is possible that kisspeptin expression in the amygdala is less in the mouse than in the rat, making it more difficult to detect the protein. This is consistent with lower ISH signals in the mouse amygdala compared to the rat (A. Kauffman, personal communication). Interestingly, the number of tdTomato positive cells in the amygdala was greater in homozygous mutant female mice than in heterozygous females (Table 3). This suggests that *Kiss1* expression in the amygdala is positively regulated by oestradiol, similar to that found in the AVPV. Indeed, it has been reported that *Kiss1* expression in the medial amygdala is increased by sex steroids in both rats and mice 28.

We used the CLARITY clearing method to make whole mouse brains transparent so that we could visualise the distribution of tdTomato‐labelled Kiss1 neurones in intact tissues without having to perform serial sections and image reconstructions. TdtTomato‐labelled cell bodies were readily visualised throughout the ventral surface of the brain. The main areas of the brain that showed kisspeptin cell bodies were the RP3V and ARC regions, as expected. Interestingly, the number of tdTomato positive neurones in the ARC was found to increase during puberty. Because the tdTomato transgene is permanently switched on after CRE‐recombination, this indicates that there is an increase in *Kiss1* promoter activity in the ARC during the pubertal transition period. This is in contrast to *in situ* hybridisation data that showed a similar number of kisspeptin positive cells in the ARC of juvenile and adult mice 29. It is possible that the *Kiss1* promoter is active prior to puberty in the ARC as indicated by the *in situ* hybridisation data but that this level of expression does not produce sufficient CRE activity to fully activate the tdTomato transgene. Increased *Kiss1* promoter activity at puberty will increase CRE expression and tdTomato activation.

Fibre projections were also visible in the CLARITY brains but whole brain imaging for fibre projections was hampered by fluorescent signal attenuation during the clearing process. As noted by Chung *et al*. 14, visualisation of fibre projections is better after immunostaining of CLARITY brains. Given that antibody staining for the whole brain takes up to 2 weeks, we applied the TDE clearing method for rapid optical clearing (within 10 h) and antibody staining of 1‐mm thick horizontal slices. Interestingly, fibres originating from the amygdala *Kiss1* neurones projected a long distance through the medial forebrain bundle to the POA and extended further into the AOB. This observation coincides with a recent study by Pineda *et al*. 27 showing amygdala kisspeptin neuronal projection to GnRH neurones in the POA and to the AOB. We also found *Kiss1* fibre projections from the ARC to several preoptic areas, including the AVPV, and also to lateral hypothalamic regions consistent with the published tracing studies 8, 9.

In summary, we have generated a Kiss‐CRE transgenic mouse that can be used to visualise *Kiss1* neurones in the brain. These mice will be useful for cell‐specific gene ablation studies or to locate *Kiss1* neurones for electrophysiological studies. They will also be useful for mapping the neuronal circuitry that regulates the reproductive axis.

## Supporting information


**Fig. S1**. Fecundity of heterozygous Kiss‐CRE mice.
**Fig. S2**. Increase in the number of tdTomato neurones in the arcuate nucleus during puberty.
