# Supplementary material for: Visualisation of Kiss1 Neurone Distribution Using a Kiss1‐CRE Transgenic Mouse
Source: J Neuroendocrinol. 2016 Oct 28;28(11):10.1111/jne.12435. doi: 10.1111/jne.12435 (PMC5091624; doi:10.1111/jne.12435)
Supplement: Supplementary file 1 — Fig. S1. Fecundity of heterozygous Kiss‐CRE mice. Fig. S2. Increase in the number of tdTomato neurones in the arcuate nucleus during puberty. [file JNE-28-0-s001.doc]

**Visualization of *Kiss1* neurone distribution using a Kiss1-CRE transgenic mouse.**

Shel Hwa-Yeo^1^*, Victoria Kyle^1^*, Paul G. Morris^1^, Sophie Jackman^1^, Lydia Sinnett-Smith, Maria Schacker^1^*, Chen Chen^2^ and William H. Colledge^1§^.

1. Reproductive Physiology Group

Department of Physiology, Development and Neuroscience

University of Cambridge

Downing Street

Cambridge

CB2 3EG

UK

2. School of Biomedical Sciences

University of Queensland

St Lucia

Brisbane

Australia

**Supporting Information**

B


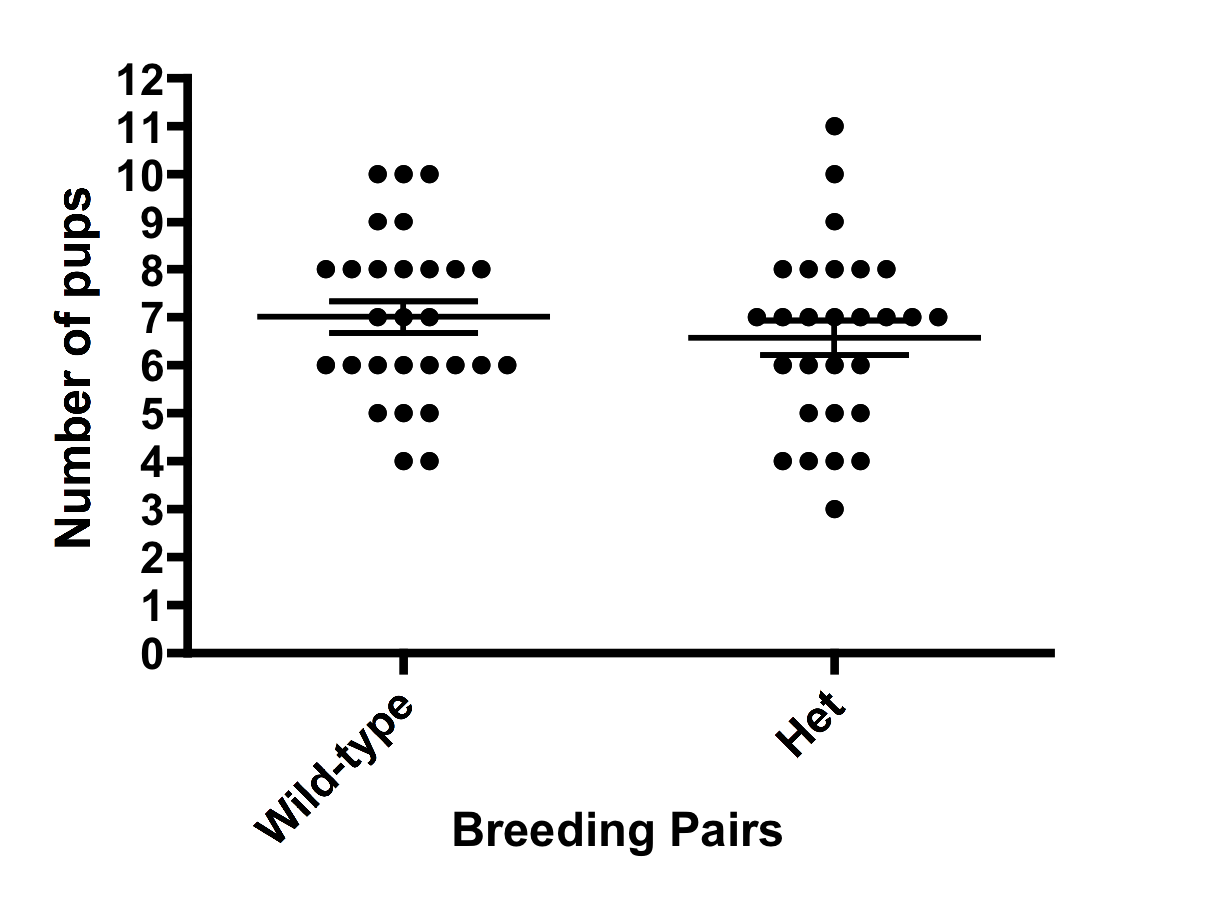


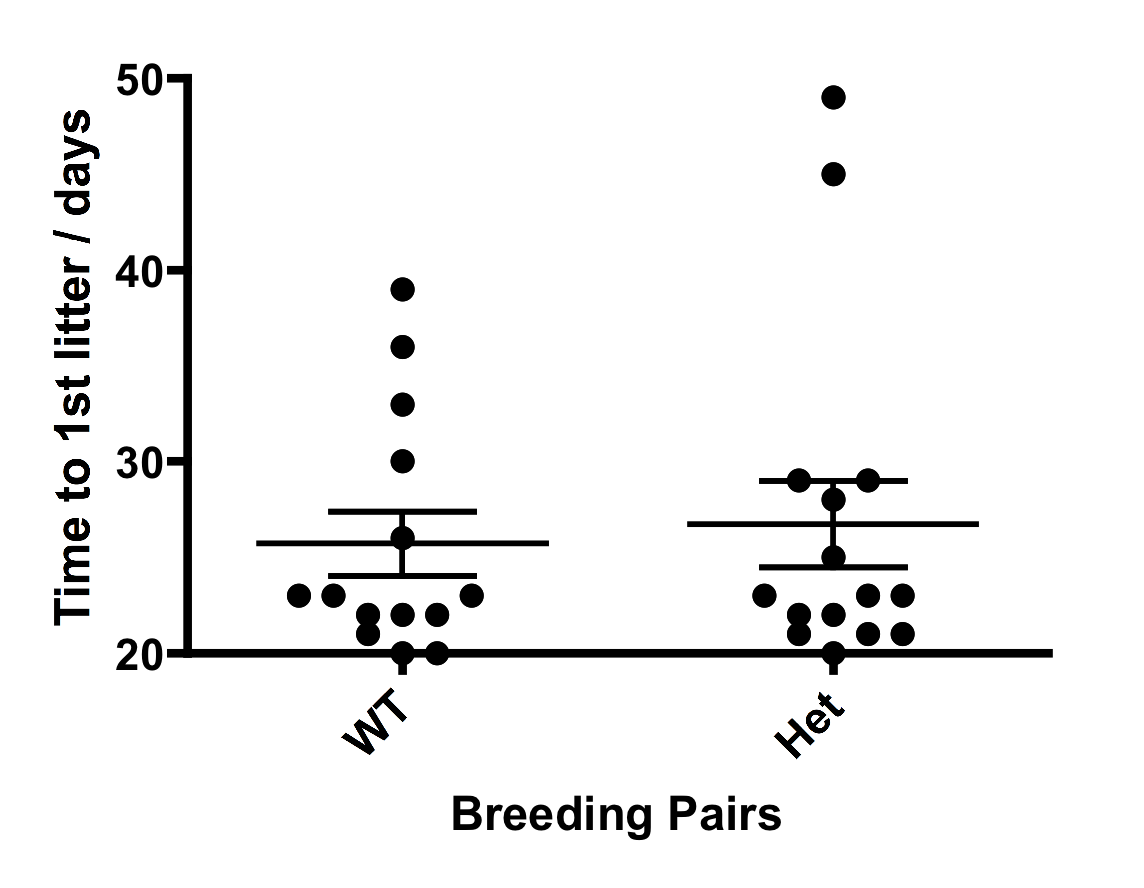


A

**Figure S1. Fecundity of Heterozygous Kiss-CRE mice.**

A. Time to 1^st^ litter for breeding pairs. Wild-type or heterozygous pairs of mice were set up for breeding and the duration to the birth of the 1^st^ litter was recorded. For wild-type mice, n=14 pairs; for heterozygous mice, n=15 pairs). There was no significant difference between the two groups (P=0.95, Mann-Whitney test).

B. Average size of litters. The number of pups born in the first 4 litters of wild-type (n=7 pairs) or heterozygous (n=7) breeding pairs was recorded. There was no significant difference between the two groups (P=0.42, Mann-Whitney test).


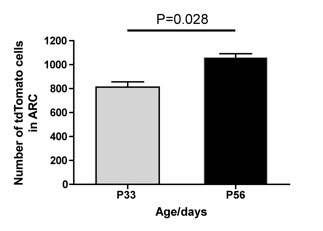


**Figure S2. Increase in the number of tdTomato neurons in the arcuate during puberty.**

Brains from Kiss-CRE:tdTomato female mice were processed by the CLARITY method to allow visualization of tdTomato neurons. Quantitation was performed on 2-D stacked images using Fuji/Image J software (n=4 brains for each age). Statistical analysis was performed using a Mann-Whitney test (2-tailed).
